# Dietary protein intake and health-related outcomes: a methodological protocol for the evidence evaluation and the outline of an evidence to decision framework underlying the evidence-based guideline of the German Nutrition Society

**DOI:** 10.1007/s00394-021-02789-5

**Published:** 2022-01-14

**Authors:** Anja Kroke, Annemarie Schmidt, Anna M. Amini, Nicole Kalotai, Andreas Lehmann, Julia Haardt, Jürgen M. Bauer, Heike A. Bischoff-Ferrari, Heiner Boeing, Sarah Egert, Sabine Ellinger, Tilman Kühn, Sandrine Louis, Stefan Lorkowski, Katharina Nimptsch, Thomas Remer, Matthias B. Schulze, Roswitha Siener, Gabriele I. Stangl, Dorothee Volkert, Armin Zittermann, Anette E. Buyken, Bernhard Watzl, Lukas Schwingshackl, the German Nutrition Society

**Affiliations:** 1 Department of Nutritional, Food and Consumer Sciences, Fulda University of Applied Sciences, Leipziger Str. 123, 36037 Fulda, Germany; 2 German Nutrition Society, Bonn, Germany; 3 Center for Geriatric Medicine and Network Aging Research, Heidelberg University, Heidelberg, Germany; 4 Department of Aging Medicine and Aging Research, University Hospital and University of Zurich, Zurich, Switzerland; 5 City Hospital Zurich-Waid, Zurich, Switzerland; 6 German Institute of Human Nutrition Potsdam-Rehbruecke, Nuthetal, Germany; 7 Institute of Nutrition and Food Science, University of Bonn, Bonn, Germany; 8 Institute for Global Food Security, Queen’s University Belfast, Belfast, UK; 9 Heidelberg Institute of Global Health, University of Heidelberg, Heidelberg, Germany; 10 Department of Physiology and Biochemistry of Nutrition, Max Rubner-Institut, Karlsruhe, Germany; 11 Institute of Nutritional Sciences, Friedrich Schiller University Jena, Jena, Germany; 12 Competence Cluster for Nutrition and Cardiovascular Health (nutriCARD) Halle-Jena-Leipzig, Jena, Germany; 13 Molecular Epidemiology Research Group, Max Delbrück Center for Molecular Medicine (MDC) in the Helmholtz Association, Berlin, Germany; 14 DONALD Study Center Dortmund, Department of Nutritional Epidemiology, Institute of Nutrition and Food Science, University of Bonn, Dortmund, Germany; 15 Department of Molecular Epidemiology, German Institute of Human Nutrition Potsdam-Rehbruecke, Nuthetal, Germany; 16 Institute of Nutritional Science, University of Potsdam, Potsdam, Germany; 17 Department of Urology, University Stone Center, University Hospital Bonn, Bonn, Germany; 18 Institute of Agricultural and Nutritional Sciences, Martin Luther University Halle-Wittenberg, Halle (Saale), Germany; 19 Institute for Biomedicine of Aging, Friedrich-Alexander-Universität Erlangen-Nürnberg, Nuremberg, Germany; 20 Clinic for Thoracic and Cardiovascular Surgery, Herz- und Diabeteszentrum Nordrhein-Westfalen, Bad Oeynhausen, Germany; 21 Institute of Nutrition, Consumption and Health, Faculty of Natural Sciences, Paderborn University, Paderborn, Germany; 22 Institute for Evidence in Medicine, Medical Center - University of Freiburg, Faculty of Medicine, University of Freiburg, Freiburg, Germany

**Keywords:** Evidence-based guideline, Protein intake, Method, Prevention, Nutrition-related diseases

## Abstract

**Purpose:**

The present work aimed to delineate (i) a revised protocol according to recent methodological developments in evidence generation, to (ii) describe its interpretation, the assessment of the overall certainty of evidence and to (iii) outline an Evidence to Decision framework for deriving an evidence-based guideline on quantitative and qualitative aspects of dietary protein intake.

**Methods:**

A methodological protocol to systematically investigate the association between dietary protein intake and several health outcomes and for deriving dietary protein intake recommendations for the primary prevention of various non-communicable diseases in the general adult population was developed.

**Results:**

The developed methodological protocol relies on umbrella reviews including systematic reviews with or without meta-analyses. Systematic literature searches in three databases will be performed for each health-related outcome. The methodological quality of all selected systematic reviews will be evaluated using a modified version of AMSTAR 2, and the outcome-specific certainty of evidence for systematic reviews with or without meta-analysis will be assessed with NutriGrade. The general outline of the Evidence to Decision framework foresees that recommendations in the derived guideline will be given based on the overall certainty of evidence as well as on additional criteria such as sustainability.

**Conclusion:**

The methodological protocol permits a systematic evaluation of published systematic reviews on dietary protein intake and its association with selected health-related outcomes. An Evidence to Decision framework will be the basis for the overall conclusions and the resulting recommendations for dietary protein intake.

**Supplementary Information:**

The online version contains supplementary material available at 10.1007/s00394-021-02789-5.

## Introduction

Traditionally, nutrition research has primarily focused on the health impact of dietary carbohydrates and dietary fats. Accordingly, previous guidelines developed by the German Nutrition Society (Deutsche Gesellschaft für Ernährung, DGE) addressed health-related recommendations for the consumption of dietary fat [1, 2] and dietary carbohydrates [3]. Today, there is also an increasing number of studies investigating the impact of dietary protein intake on health-related outcomes, allowing the establishment of an evidence-based guideline on dietary protein intake. This upcoming guideline will focus on the role of dietary protein intake for the primary prevention and risk modification of various non-communicable diseases. Upon starting the development process for the evidence-based guideline on dietary protein intake, the existing methodology applied for the previous guidelines of the DGE had to be adapted to recent developments in the field. These novel aspects relate to the availability of topical evidence evaluation tools and the increased availability of systematic reviews (SRs) with or without meta-analyses (MA). A further novelty of the revised methodology includes new working principles, such as the four-eye principle and the outline of an Evidence to Decision (EtD) framework [4, 5]. Accordingly, the objective of the current protocol was to develop a revised methodological procedure for the systematic literature reviews and for the deduction of an evidence-based guideline for dietary protein intake. The key question to be addressed in this guideline to assess the overall certainty of evidence was defined as follows: Does dietary protein intake with regard to quantitative (higher vs. lower dietary protein intake) and qualitative considerations (total, plant-based, animal-based or supplemental protein intake) affect the development of selected health-related outcomes (i.e. blood pressure, body weight and other body weight-related outcomes, bone health, cancer, cardiovascular diseases, kidney health, muscle health, type 2 diabetes mellitus, inflammatory bowel disease) in the general adult population? For addressing this research question, this publication therefore describes in detail the novel methodological procedures of the literature reviews for the assessment of the overall certainty of evidence. The following steps for the derivation of summary conclusions and recommendations based on an EtD framework will only be outlined in general. Corresponding details will be published separately.

## Methods

A guideline panel consisting of DGE staff members, DGE scientific board members and selected experts was constituted in 2016 to develop the evidence-based guideline on dietary protein intake. This panel selected a panel coordinator (BW) and reached a consensus-based decision on the outcomes to be addressed in the guideline, taking aspects, such as disease burden and expected relevance of protein intake, into account. Since the amount of protein reported in observational studies or used in intervention studies varied notably, the panel refrained from a strict unifying definition for high or low protein intake (such as above or below 0.8 g/kg body weight per day). Instead, within each chapter, the link of higher and lower protein intake as reported or used in the included studies to the specific endpoint will be evaluated. A methodology task force (AK, AEB, LS) was set up to delineate the procedures to be followed for establishing the guideline. A working group (AS, AMA, NK, AL, JH) was commissioned to perform the literature search and selection, data extraction and assessment of methodological quality, and outcome-specific certainty of evidence. Conflict of interest statements were obtained from all members of the guideline panel and published (Supplement 1).

In consideration of the increased availability of SRs with or without MA, it was primarily decided to develop an approach based on systematic searches for these study types and to perform umbrella reviews for each of the nine selected health-related outcomes. A continuous publication of each successively completed umbrella review, from now on referred to as guideline chapter, and a final overall recommendation based on these guideline chapter conclusions, were set out to be the backbone for the protein guideline. The evidence-based guideline on dietary protein intake was registered as an umbrella review in PROSPERO (CRD42018082395) [6].

## Results

**Fig. 1:**
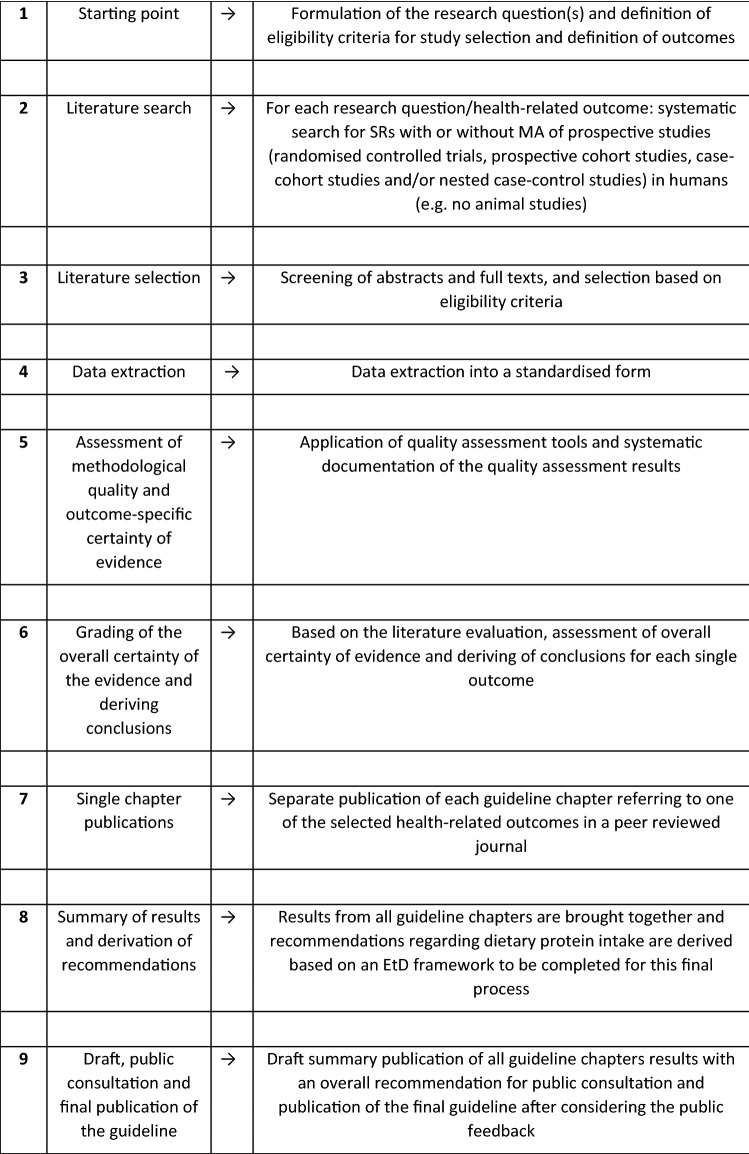
Nine-step scheme for developing the evidence-based guideline for protein intake of the German Nutrition Society. *EtD* Evidence to Decision framework, *MA* meta-analysis, *SR* systematic review

Like the previous approaches to derive the guidelines of the DGE [7], a multi-step scheme consisting of nine steps constitutes the backbone for the methodological procedures. Figure 1 presents this nine-step scheme for the development of the DGE’s evidence-based guideline for dietary protein intake. Hereafter, each single step is outlined in detail.

### Step 1: starting point

Explicit research questions for each guideline chapter are defined. To support the formulation of explicit research questions, the PICO scheme (population, intervention, comparator, outcome) or its modification, the PECO scheme (population, exposure, comparator, outcome), was applied [8]. Table 1 presents the scheme and the criteria derived from it for eligible SRs with or without MA.

**Table 1:** PICO(ST), or PECO(ST) scheme, respectively, for the definition of study eligibility criteria for literature selection

	Category	Inclusion criteria	Exclusion criteria
P	Population	• General adult population (≥ 18 years) • Including older adults and recreational athletes • Including people with overweight, obesity, (pre)hypertension and abnormal blood lipids	• Infants, children, adolescents • Pregnant or breastfeeding women • Top athletes
I/E	Intervention or exposure^a^	• Higher protein intake • Intake of total protein • Intake of plant protein • Intake of animal protein • Intake of protein supplements	• Protein was not specifically investigated (e.g. whole food approaches) • Peptides and/or amino acids
C	Comparator	• Lower protein intake • Other type of protein intake • Placebo	
O	Outcomes^b,c^	• Blood pressure • Body weight and other body weight-related outcomes • Bone health • Cancer • Cardiovascular diseases • Type 2 diabetes mellitus • Kidney health • Muscle health	
S	Study design	• SR with or without MA of prospective studies (RCTs, prospective cohort studies, case-cohort studies and/or nested case-control studies)^d^	• Individual studies: RCTs, prospective cohort studies, other primary studies • SR of only case-control studies or cross-sectional studies, case studies • Umbrella reviews
T	Time	• Any study duration	

*MA* meta-analysis, *RCT* randomised controlled trial, *SR* systematic review

^a^The categories will be used as defined by the authors of the SR

^b^More explicit definitions will be provided in the respective outcome-specific guideline chapters

^c^Outcomes are eligible as categorical (e.g. incidence of obesity) as well as continuous (e.g. change of body weight) variables

^d^Case-control studies are tolerated if prospective studies are predominant (> 50% of all studies) in the respective SR

### Step 2: literature search

For the systematic literature searches, the databases PubMed, Cochrane Database of Systematic Reviews, and Embase are used. Due to restricted resources, the outcome-specific searches are performed successively, covering a 10-year period. Details on search periods and search updates will be presented in the respective outcome-specific guideline chapters. Specific search terms were selected, and search strings were formulated. Table 2 presents the applied search terms addressing SRs and protein exposure type.

**Table 2:** Search terms for systematic literature searches

Research topic	Database		
	PubMed	Cochrane	Embase
Systematic reviews^a^	Meta-analy* [tiab] OR "meta-analysis" [tiab] OR "meta analyses" [tiab] OR "meta analysis" [tiab] OR metaanalysis [tiab] OR "meta-analyze" [tiab] OR "meta-analysis" [Publication Type] OR systematic [sb] OR "systematic review" [tiab]	–	'Meta analysis'/exp OR 'systematic review'/exp OR Meta-analy*:ti,ab OR 'meta-analysis':ti,ab OR 'meta analyses':ti,ab OR 'meta analysis':ti,ab OR metaanalysis:ti,ab OR 'meta-analyze':ti,ab OR 'systematic review':ti,ab
Protein	"dietary proteins" [mh] OR "diet, protein-restricted"[mh] OR "whey proteins" [mh] protein[tiab] OR proteins[tiab] OR "high-protein" [tiab]OR "low-protein" [tiab] OR "wheypowder" [tiab] OR "wheypowders" [tiab] OR "hypoprotein diet" [tiab] OR "Peptidylgroup" [tiab] OR "Dairy product" [tiab] OR "dairy products" [tiab]OR "protein-free" [tiab] OR "protein-restricted" [tiab]	[mh "dietary proteins"] OR [mh "diet, protein-restricted"] OR [mh "whey proteins"] OR protein:ti,ab OR proteins:ti,ab OR "high-protein":ti,ab OR "low-protein":ti,ab OR "whey powder":ti,ab OR "whey powders":ti,ab OR "hypoprotein diet":ti,ab OR "peptidyl group":ti,ab OR "dairy product":ti,ab OR "dairy products":ti,ab OR "protein-free":ti,ab OR "protein-restricted":ti,ab	'protein intake'/exp OR 'protein restriction'/exp OR 'dairy product'/exp OR 'yolk protein'/exp OR 'proteins by anatomical concept'/exp OR 'proteins by organism'/exp OR protein:ti,ab OR proteins:ti,ab OR 'high-protein':ti,ab OR 'low-protein':ti,ab OR 'whey powder':ti,ab OR 'whey powders':ti,ab OR 'hypoprotein diet':ti,ab OR 'peptidyl group':ti,ab OR 'dairy product':ti,ab OR 'dairy products':ti,ab OR 'protein-free':ti,ab OR 'protein-restricted':ti,ab

Outcome-specific search terms will be provided in the respective guideline chapters

^a^PubMed changed the search strategy of its [sb]-filter to identify systematic reviews in 01/2019. To maintain continuity, we used this previous version for all our literature searches

Literature search is restricted to articles published in English or German within the last ten years. The search period is extended if update searches are performed. SRs with or without MA of prospective studies (randomised controlled trials (RCTs), prospective cohort studies, case-cohort studies, or nested case–control studies) are eligible. Those study designs are considered the most reliable in nutrition research [9]. SRs also considering case–control studies are only included if prospective studies are predominant (> 50% of all studies).

### Step 3: literature selection

Selection of SRs is performed by two authors independently according to the predefined inclusion and exclusion criteria (Table 1). Any disagreement is resolved by the authors via discussion and consensus, if necessary, with consultation of the methodology task force. Title and/or abstract of retrieved studies are screened to identify potentially eligible studies. The full texts of these records are retrieved and assessed for eligibility. Moreover, the reference lists of the included SRs are searched manually for additional publications. As with previous guidelines of the DGE, it is tolerated that some of the primary studies were incorporated more than once into different SRs of the same outcome.

Flow diagrams in each guideline chapter will outline the study selection process, including the number of records identified, the number of records considered as potentially relevant after title/abstract screening, and finally the number of records included and excluded, respectively, with reasons for excluding records following full-text screening.

### Step 4: data extraction

Two authors independently extract the following data from each included SR using a standardised form: first author of the SR, year of publication, study type of relevant primary studies, study period of relevant primary studies, study population of relevant primary studies, dietary assessment method of relevant primary studies, range of protein intake if provided, intervention/exposure(s) of primary studies, outcome(s) investigated by primary studies, effect estimates including 95% CI, *p* values, heterogeneity estimates and subgroup analyses. Any discrepancies between the two authors are resolved through discussion, if necessary, with consultation of the methodology task force. In addition, an overview of all primary sources included in the SRs on the respective health outcome is provided to highlight overlaps.

### Step 5: assessment of methodological quality and outcome-specific certainty of evidence

For the assessment of methodological quality of the retrieved SRs, a modified version of the “*A Measurement Tool to Assess Systematic Reviews 2*” tool (AMSTAR 2) [10] is used (Supplement 2). The description of the applied modifications of AMSTAR 2, and the rationale for such modifications are summarised in Supplement 3, additional to the original AMSTAR 2 questionnaire. The modified version contains 14 items evaluating the quality of the SR, addressing the risk of bias assessment, the quality of statistical analyses and reporting of results and transparency of potential sources of conflict of each SR. The SRs are rated on a scale from high quality to critically low quality according to the existence of critical and non-critical methodological weaknesses. Assessment of the methodological quality is performed for each included SR.

The NutriGrade scoring tool is used to assess the outcome-specific certainty of evidence for each SR with at least one MA of RCTs and/or prospective cohort studies [11] (Supplement 4). The outcome-specific certainty of evidence derived from each SR without MA of RCTs and/or prospective cohort studies is rated with an adapted version of NutriGrade (Supplement 5). NutriGrade aims to assess the certainty of evidence of an association or effect between different dietary factors and outcomes, taking into account nutrition research-specific requirements not considered by other tools. An important novelty of NutriGrade was the modified classification for MA of RCTs and cohort studies compared with the traditional GRADE approach (initially classifying RCTs with a high score and cohort studies with a low score) [11, 12]. Meanwhile, the GRADE approach was amended in a way that cohort studies can now also be assigned an initially high score, when risk of bias tools such as ROBINS-I are used [13]. The NutriGrade scoring tool utilises a numerical scoring system with a maximum score of 10 points. Seven items for SRs with MA of RCTs and eight items for MA of prospective cohort studies evaluate risk of bias/study quality/limitations, precision, statistical heterogeneity, directness, publication bias, funding bias, study design (only for SRs with MA of RCTs), magnitude of effect size, and dose–response analysis (both only for SRs with MA of prospective cohort studies) (Supplement 4). Based on the scoring system, four categories rate the potential outcome-specific certainty of evidence reaching from high (≥ 8 points) to moderate (6 to < 8 points), to low (4 to < 6 points) and very low (0 to < 4 points) (Supplement 4). As described above, the slight adaptions for the procedure for SRs without MA are described in detail in Supplement 5. The assessment of the certainty of evidence is performed on an outcome basis for each SR with or without MA separately. If a SR with or without MA reports more than one relevant outcome, each outcome-specific certainty of evidence is assessed separately.

Two authors perform the assessments of methodological quality and of outcome-specific certainty of evidence independently. Any inconsistencies in the rating are resolved by discussion, if necessary, with consultation of the methodology task force.

SRs rated as “critically low” by AMSTAR 2 will not be considered. The respective information on these exclusions will be given in each guideline chapter.

### Step 6: rating of the overall certainty of the evidence and deriving conclusions

The rating of the overall certainty of evidence is assessed separately for each relevant exposure–outcome association considering all relevant SRs. The overall rating ranges from convincing, probable, possible to insufficient. The first step assesses whether there is at least one SR with or without MA of prospective studies. If more than one SR with or without MA is available, all (convincing) or the majority (probable, possible) of the results must be consistent. Biological plausibility must be given in any case (direct or inverse association). In the final step, the results of the NutriGrade and AMSTAR 2 ratings are considered. Depending on the level of evidence, the SRs must have achieved a certain rating in both tools. If no SR is identified or if the majority of SR reached a very low outcome-specific certainty of evidence and/or a low methodological quality, the overall certainty of evidence is considered insufficient. The detailed underlying criteria are outlined in Table 3. Two authors make suggestions for rating the overall certainty of evidence. Any discrepancies between the two authors are identified and resolved through discussion, if necessary, with consultation of the methodology task force.

**Table 3:** Grading the overall certainty of evidence according to methodological quality, outcome-specific certainty of evidence, biological plausibility and consistency of results, and definition of the overall certainty of evidence in a modified form according to the GRADE approach [11]

Overall certainty of evidence	Underlying criteria	Definition/Explanation
Convincing	• At least one SR with or without MA of prospective studies available • If more than one SR with or without MA are available: all overall results must be consistent.^a^ • In case of a positive or negative association, biological plausibility is given • All included SRs with or without MA must reach at least a “moderate” outcome-specific certainty of evidence^b^; in addition all included SRs must reach at least a methodological quality^c^ of “moderate”	There is high level of confidence that the true effect lies close to that of the estimate(s) of the effect
Probable	• At least one SR with or without MA of prospective studies available • If more than one SR with or without MA are available, the majority of overall results must be consistent.^a^ • In case of a positive or negative association, biological plausibility is given • The majority^d^ of included SRs with or without MA must have reached at least a “moderate” certainty of evidence^b^; in addition all included SRs must reach at least a methodological quality^c^ of “moderate”	There is moderate confidence in the effect estimate(s): The true effect is likely to be close to the estimate of the effect, but there is a possibility that it is substantially different
Possible	• At least one SR with or without MA of prospective studies available • If more than one SR with or without MA are available, the majority of overall results must be consistent.^a^ • In case of a positive or negative association, biological plausibility is given • The majority^d^ of included SRs with or without MA must reach at least a “low” certainty of evidence^b^; in addition the majority^d^ of all included SRs must reach at least a methodological quality^c^ of “moderate”	Confidence in the effect estimate(s) is limited: The true effect may be substantially different from the estimate of the effect
Insufficient	• No SR is available *OR* • The majority^d^ of included SRs with or without MA reach a “very low” certainty of evidence^b^; in addition the majority of all included SRs reach a methodological quality^c^ of “low”	There is very little confidence in the effect estimate (s): The true effect is likely to be substantially different from the estimate of effect

*MA* meta-analysis, *SR* systematic review

^a^Consistent = overall results of the SR have to be consistently either risk reducing or risk elevating or consistently showing no risk association

^b^Outcome-specific certainty of evidence refers to the NutriGrade rating

^c^Methodological quality refers to the AMSTAR 2 rating**;** SRs rated as “critically low” by AMSTAR 2 are not considered

^d^Majority: > 50% of the included SRs

The suggestions of the overall certainty of evidence rating, and the outcome-specific conclusions will be finalised after discussing them with all guideline panel members to reach an overall expert decision.

### Step 7: single guideline chapter publications

The finalised guideline chapters, e.g. the outcome-specific umbrella reviews, will be submitted for publication to an international journal with peer review. In case of insufficient overall evidence regarding a selected outcome, an umbrella review might not be possible and is therefore not submitted for publication.

### Step 8: summary of results and derivation of recommendations

Once all guideline chapters will be finalised, the guideline panel will reconvene to summarise all results and conclude comprehensive recommendations. To that end, the guideline panel will adapt and modify the GRADE EtD frameworks [5] as a systematic and transparent approach to derive overall recommendations [5, 14]. Following the EtD principle of formulating questions, making assessments, and drawing conclusions, additional criteria (see below) will be addressed by providing the respective best available evidence, complemented by additional information or considerations. Based on this information, the guideline panel will make informed judgements regarding each criterion. Both information on underlying evidence used and on judgements when evidence was lacking will be documented. Finally, the guideline panel will draw a conclusion about the direction (for, or against or no recommendation) and strength (strong or conditional) of their recommendation. A justification for the recommendation will be provided.

To derive these recommendations, the panel will judge and consider the following criteria, supported by the best available evidence. More details on the following aspects 2 to 7 will be provided in a separate publication:

1. Overall certainty of evidence: as described above [14].
2. Problem priority: weighting of outcomes (according to epidemiological data on outcome frequency and severity indicators) to prioritise potentially contradictory outcome-specific results [14].
3. Weighting of benefits and harms: e.g. for some outcomes increased intake of protein might be beneficial but for others rather harmful. Therefore, it will be necessary to weight both sides.
4. Dietary intake levels and preferences: dietary habits, cultural preferences in food intake, and current protein intake levels of the target populations (adult general population living in Germany).
5. Ecological sustainability: environmental impact (e.g. greenhouse gas emissions, land use, freshwater, etc.) and economic considerations (e.g. financial costs of recommendations) [15, 16].
6. Acceptability and feasibility of recommendation: estimate of possible implementation impact, including potential barriers [14].
7. Potential impacts on health equity: considering differential effects on disadvantaged populations or specific population subgroups.

### Step 9: draft, public consultation, and final publication of the guideline

A draft version of the protein guideline will be published online for public consultation. Comments will be accepted for a period of two months. After careful consideration of all the remarks by the entire guideline panel, the final guideline will be published.

## Discussion

Today, an overwhelming body of nutrition research study data is available which can hardly be critically evaluated and made applicable by a single user, be it a research scientist or a practitioner. The systematically developed and evidence-informed dietary guidelines of the DGE should provide a basis to assist nutrition experts as well as other experts to make appropriate professional decisions in their respective fields. Similar endeavours have been described by methodologists, pointing out the need to provide trustworthy nutrition guidelines by adhering to internationally accepted and transparently described literature review standards [17].

The upcoming guideline for dietary protein will reflect the current knowledge on quantity and quality of dietary protein intake and the risk association with several health-related outcomes (blood pressure, body weight and body weight-related outcomes, bone health, cancer, cardiovascular diseases, kidney health, muscle health, inflammatory bowel disease and type 2 diabetes mellitus). Importantly, in addition to the overall certainty of evidence, several additional aspects will have been considered in the overall recommendations on dietary protein intake, including considerations on problem priority, benefit and harms, dietary intake levels and preferences, ecological sustainability of a certain protein intake level and financial costs of recommendations, acceptability, feasibility, and potential impacts on health equity. Finally, the results of an open consultation will be handled before the final guideline on protein intake is published. To that end, this publication described in detail the overview on the procedures to assess the overall certainty of evidence, and outlined cornerstone aspects of an EtD framework that is to be adapted to the current GRADE EtD frameworks [14]. The GRADE EtD framework is a comprehensive and rigorous guideline approach that is considered to strengthen transparency, trust, and credibility of resulting guidelines [12]. Once finalised and applied, the EtD framework allows to inform about judgements made, including the evidence supporting those judgements [5]. By providing this detailed methodological description the necessity and demanded transparency of such processes [17] is assured.

The multi-step procedure followed in previous guidelines of the DGE (e.g., relating to carbohydrate [3] and fat intake [1, 2]) had to be adapted according to recent developments in the field of evidence synthesis and interpretation. In contrast to the previous guidelines of the DGE, the current approach is based on umbrella reviews while primary studies will not be included. We therefore had to adjust the method for the quality assessment of the included SRs. AMSTAR 2 [10] and NutriGrade [11] are applied to assess the methodological quality and outcome-specific certainty of evidence, respectively. Grading the outcome-specific certainty of evidence is considered of fundamental importance as it can improve the trustworthiness of findings [12]. For grading the overall certainty of evidence, a scheme was developed, accommodating nutrition research-specific aspects, including for example “biological plausibility”. These criteria extend the scheme used in previous guidelines of the DGE and are now adapted to the newly applied study quality and certainty assessments. By setting the criteria to be fulfilled for classifying the certainty of evidence into one of four categories, a standardised procedure for deriving conclusions on the relation between dietary protein intake and each of the addressed disease outcomes was strived for.

After finalising all umbrella reviews, the final steps of the guideline development will be entered by completing the EtD framework: all derived conclusions of the single, disease-specific umbrella reviews are to be summarised by weighting their relevance for the target population according to epidemiological, clinical, public health and/or ecological aspects, and to formulate overall recommendations. As this step will still take some time to be performed, only cornerstones of this process have been presented here and details are still to be developed and then published. This will allow to rely on the most recent developments in this field.

The strengths of this methodological protocol include a commitment to internationally accepted guideline development standards, such as performing the literature search in different databases, consideration of evidence from SRs with or without MA, evaluation of the certainty of evidence and use of an EtD framework (including aspects of sustainability). Adhering to these standards will ensure that the derived recommendations will be based on a high quality, novel overview of systematic literature reviews. Nevertheless, we are aware that including solely SRs with or without MA involves the potential risk of overlooking evidence from (recently published) primary studies. When setting up the methodology, we assumed that for the majority of guideline chapters the amount and nature of SRs will likely be sufficient to conduct umbrella reviews and that they will be sufficiently up to date.

The dietary recommendations derived in the guideline of the DGE on protein intake will be put forward by a group of experts in nutritional physiology, public health and clinical nutrition, nutritional epidemiology, and evidence synthesis methods. Respective details from each member of the guideline working group are made available (Supplement 1), thereby disclosing potential conflicts of interest.

The described methodological procedure has various limitations. The most relevant general aspect to be discussed refers to the predominantly available non-randomised studies, e.g. prospective observational studies, which have been repeatedly criticised to be prone to various biases and (residual) confounding [12]. To address this, vigorous application of respective assessment tools has been recommended [12]. Here, AMSTAR 2 is used for the methodological quality assessment of the retrieved SRs including risk of bias assessment, and NutriGrade to evaluate the outcome-specific certainty of evidence for each SR, also addressing risk of bias. It has, however, to be conceded that the guideline panel decided to evaluate the methodological SR quality using a modified version of AMSTAR 2 and to combine it with the outcome-specific certainty of evidence assessment/rating to obtain a complete picture of the quality of the included publications. We are aware that the approach to incorporate the methodological quality of SRs into the overall evidence has been criticised by the GRADE working group [18], as it was argued that it is problematic and misleading to include the quality of a SR as a factor to determine the certainty of a body of evidence. We thus follow the proposal of this working group to exclude low-quality SRs (better called a non-credible SR) from the overview of reviews [18]. In addition to the necessity of such assessments, it has been shown that a separation of the certainty of the evidence assessment on the one hand from confidence in recommendations on the other hand enhances clarity of proceedings and strengthens credibility of dietary recommendations although the certainty of evidence itself is not increased [12]. Such a separation is part of the here described procedure. A further limitation might be seen in the modification of answer options, e.g. of the original question 7. To abstain from demanding an explicit list of studies with exclusion reason while a flowchart is available is considered to be a pragmatic decision, given the available SRs and the rather conservative overall certainty rating.

Furthermore, both SRs and umbrella reviews as such hold limitations. Although SRs potentially provide stronger evidence than individual studies alone, methodological and statistical heterogeneity of the included studies represent, among others, difficult to solve challenges. In terms of fundamental approaches to umbrella reviews, instead of searching for the various SRs available, only the best or latest could be considered. Given the considerable overlap of SRs on a specific topic, this could be an option. However, since we aim for a comprehensive summary of the current body of SR evidence, it is preferred to include all SRs regardless of overlap. This decision is supported by the recently revised chapter of the Cochrane handbook “Overview of Reviews” [19].

## Conclusion

A comprehensive protocol for systematic literature reviews relying on umbrella reviews and cornerstones of an EtD framework has been developed for the derivation of the upcoming evidence-based guideline for dietary protein intake of the DGE. Once the recommendations of the guideline are derived and published, nutrition and health experts as well as public health policymakers may profit from these guidelines; its implications include a better understanding of the desirable and undesirable associations/effects of protein consumption and provide consequently the option to promote a better-informed decision-making by the general population.

## Supplementary Information

Below is the link to the electronic supplementary material.

Supplementary file1 (XLSX 14 KB)

Supplementary file2 (PDF 405 KB)

Supplementary file3 (PDF 267 KB)

Supplementary file4 (PDF 316 KB)

Supplementary file5 (PDF 293 KB)

## Data Availability

Not applicable.

## Abbreviations

AMSTAR 2: A Measurement Tool to Assess Systematic Reviews 2
DGE: Deutsche Gesellschaft für Ernährung (German Nutrition Society)
MA: Meta-analysis/meta-analyses
RCT: Randomised controlled trial
SR: Systematic review
EtD: Evidence to Decision
