# Supplementary material for: Dietary protein intake and health-related outcomes: a methodological protocol for the evidence evaluation and the outline of an evidence to decision framework underlying the evidence-based guideline of the German Nutrition Society
Source: Eur J Nutr. 2022 Jan 14;61(4):2091–101. doi: 10.1007/s00394-021-02789-5 (PMC9106629; doi:10.1007/s00394-021-02789-5)
Supplement: Supplementary file 4 — Supplementary file4 (PDF 316 KB) [file 394_2021_2789_MOESM4_ESM.pdf]

Supplement 4. NutriGrade scoring tool for SRs with MA.

This supplement provides an overview of the applied NutriGrade scoring system. Detailed guidance and information on the allocation of points can be found here: Schwingshackl L, Knüppel S, Schwedhelm C, Hoffmann G, Missbach B, Stelmach-Mardas M, Dietrich S, Eichelmann F, Kontopanteils E, Iqbal K, Aleksandrova K, Lorkowski S, Leitzmann MF, Kroke A, Boeing H: Perspective: NutriGrade: A scoring system to assess and judge the meta-evidence of randomized controlled trials and cohort studies in nutrition research. Adv Nutr 2016;7:994–1004.

NutriGrade scoring system for SRs with MA of RCTs

1) Risk of bias/ study quality/ study limitations **(3 P)**
   a. No quantitative and descriptive information available (0 P)
   b. Risk of bias (3 P)
      i. Sequence generation[1]
      ii. Allocation concealment[1]
      iii. Blinding of participants and personnel[1]
      iv. Blinding of outcome assessment personnel[1]
      v. Incomplete outcome[1]
      vi. Selective reporting[1]
   c. Study quality (2 P)[2]

2) Precision **(1 P)**
   a. <400 participants OR 400-2000 participants, but 95% CI overlaps the null value (0 P)
   b. >2000 participants OR 400-2000 participants, but 95% CI excludes the null value (1 P)

3) Heterogeneity **(1 P)**
   a. ≤ 5 studies (0 P)
   b. 6-9 studies (if ≥10 studies; multiply points by 2):
      i. I^2 (H^2 and/or tau^2) (0.1 P)
      ii. CIs for I^2 (0.1 P)
      iii. If I^2 <40% (0.3 P) skip **iv**
      iv. Modelling detected heterogeneity (I^2≥40%) with random effects model (0.1 P)
         1. Exploring detected heterogeneity with subgroup analysis or meta-regression (0.1 P)
         2. Sensitivity analyses with higher levels of heterogeneity (0.1 P)

4) Directness **(1 P)**
   a. Differences in population; differences in intervention; surrogate markers; network meta-analysis (0 P)
   b. No important differences in population or intervention; hard clinical outcome (1 P)

5) Publication bias **(1 P)**
   a. <5 studies OR evidence for severe bias with test or plot OR publication bias not assessed (0 P)
   b. No evidence for publication bias with test or plot (5-9 studies) OR evidence for moderate/small amount of publication bias with test or plot (0.5 P)
   c. No evidence for publication bias with test or plot (≥10 studies) (1 P)

6) Funding bias **(1 P)**
   a. Industry funding OR conflict of interest (0 P)
   b. Private institutions, foundations, non-governmental organizations (0.5 P)
   c. Academic institutions, research institutions (1 P)

2

7) Study design **(+ 2 P)**

Overall Score[3]

P: point(s); RCT: randomized controlled trial.

[1] ≥2/3 of studies low risk of bias = 0.5 P; >1/3 of studies high risk of bias OR not assessed = 0 P; unclear risk of bias = 0.25P)

[2] ≥2/3 of overall score = 2 P; ≥1/3 of overall score = 1 P; otherwise = 0 P

[3] 0-3.99: very low meta-evidence; 4-5.99: low meta-evidence; 6-7.99: moderate meta-evidence; ≥8: high meta-evidence

NutriGrade scoring system for SRs with MA of cohort studies

1) Risk of bias/ study quality/ study limitations **(2 P)**
   a. No information available (0 P)
   b. Risk of bias (2 P)
      i. Ascertainment of exposure[1]
      ii. Adjusted basic & outcome relevant model[1]
      iii. Assessment of outcome[1]
      iv. Adequacy of follow-up duration[1]
   c. Study quality (2 P)[2]
2) Precision **(1 P)**
   a. <500 events OR ≥500 events but 95% CI overlaps the null, and includes important benefit (RR: <0.8) or harm (RR: >1.2) (0 P)
   b. ≥500 events and the 95% CI excludes the null values; ≥500 events but 95% CI overlaps the null, and excludes important benefit (RR: <0.8) or harm (RR: >1.2) (1 P)
3) Heterogeneity **(1 P)**
   a. ≤ 5 studies (0 P)
   b. 6-9 studies (if ≥10 studies; multiply by 2):
      i. I^2 (H^2 and/or tau^2) (0.1 P)
      ii. CIs for I^2 (0.1 P)
      iii. If I^2 <40% (0.3 P) skip iv
      iv. Modelling detected heterogeneity (I^2 ≥40%) with random effects model (0.1 P)
         1. Exploring detected heterogeneity with subgroup analysis or meta-regression (0.1 P)
         2. Sensitivity analyses with higher levels of heterogeneity (0.1 P)
4) Directness **(1 P)**
   a. Differences in population; differences in intervention; surrogate markers; network meta-analysis (0 P)
   b. No important differences in population or intervention; hard clinical outcome (1 P)
5) Publication bias **(1 P)**
   a. <5 studies OR evidence for severe bias with test or plot OR publication bias not assessed (0 P)
   b. No evidence for publication bias with test or plot (5-9 studies) OR evidence for moderate/small amount of publication bias with test or plot (0.5 P)
   c. No evidence for publication bias with test or plot (≥10 studies) (1 P)
6) Funding bias **(1 P)**
   a. Industry funding OR conflict of interest (0 P)
   b. Private institutions, foundations, non-governmental organizations (0.5 P)
   c. Academic institutions, research institutions (1 P)
7) Effect size **(2 P)**
   a. No effect (HR/RR: 0.80-1.20) (0 P)
   b. Moderate effect size (HR/RR: <0.80-0.50 or >1.2-2.00) (1 P)
   c. Large effect size (HR/RR: <0.50 or >2.00) (2 P)
8) Dose-response **(1 P)**
   a. No dose-response relationship (corresponding statistical test non- significant) (0 P)
   b. Linear and/ or non-linear dose-response relationship (corresponding statistical test significant) (1 P)

Overall Score[3]

P: point(s); RR: risk ratio.
[1] ≥2/3 of studies low risk of bias = 0.5 P; >1/3 of studies high risk of bias OR not assessed = 0 P; unclear risk of bias = 0.25 P)
[2] cut-off for different quality scale (≥3/4 of overall score= 2 P; ≥1/2 of overall score= 1 P; <1/2 of overall score= 0 P); i.e. **Newcastle-Ottawa Scale** (mean): ≥7= 2 P; 4-6.9= 1 P; 0-3.9= 0 P;
[3] 0-3.99: very low meta-evidence; 4-5.99: low meta-evidence; 6-7.99: moderate meta-evidence; ≥8: high meta-evidence
