# Supplementary material for: Dietary protein intake and health-related outcomes: a methodological protocol for the evidence evaluation and the outline of an evidence to decision framework underlying the evidence-based guideline of the German Nutrition Society
Source: Eur J Nutr. 2022 Jan 14;61(4):2091–101. doi: 10.1007/s00394-021-02789-5 (PMC9106629; doi:10.1007/s00394-021-02789-5)
Supplement: Supplementary file 5 — Supplementary file5 (PDF 293 KB) [file 394_2021_2789_MOESM5_ESM.pdf]

Supplement 5. NutriGrade scoring tool for SRs without MA

This supplement provides an overview of the applied NutriGrade scoring system. Detailed guidance and information on the allocation of points can be found in: Schwingshackl L, Knüppel S, Schwedhelm C, Hoffmann G, Missbach B, Stelmach-Mardas M, Dietrich S, Eichelmann F, Kontopanteils E, Iqbal K, Aleksandrova K, Lorkowski S, Leitzmann MF, Kroke A, Boeing H: Perspective: NutriGrade: A scoring system to assess and judge the meta-evidence of randomized controlled trials and cohort studies in nutrition research. Adv Nutr 2016;7:994–1004.

NutriGrade scoring system for SRs without MA of RCTs

1) Risk of bias/ study quality/ study limitations **(3 P)** [ ]
   a. No quantitative and descriptive information available (0 P)
   b. Risk of bias (3 P)
      i. Sequence generation[1]
      ii. Allocation concealment[1]
      iii. Blinding of participants and personnel[1]
      iv. Blinding of outcome assessment personnel[1]
      v. Incomplete outcome[1]
      vi. Selective reporting[1]
   c. Study quality (2 P)[2]

2) Precision **(1 P)** [ ]
   a. <400 participants (0 P)
   b. ≥400 participants (1 P)

3) Heterogeneity **(1 P)** [ ]
   a. >1/3 of included studies have an inconsistent result (i.e. point estimates and/or 95% CI did not overlap between studies) (0 P)
   b. ≥2/3 of included studies have a consistent result (i.e. point estimates and/or 95% CI did overlap between studies) (1 P)

4) Directness **(1 P)** [ ]
   a. Differences in population; differences in intervention; surrogate markers; network meta-analysis (0 P)
   b. No important differences in population or intervention; hard clinical outcome (1 P)

5) Funding bias **(1 P)** [ ]
   a. Industry funding OR conflict of interest (0 P)
   b. Private institutions, foundations, non-governmental organizations (0.5 P)
   c. Academic institutions, research institutions (1 P)

6) Study design **(+ 2 P)** [2]

Overall Score[3] [ ]

95% CI: confidence intervals; P: point(s); RCT: randomized controlled trial.
[1] ≥2/3 of studies low risk of bias = 0.5 P; >1/3 of studies high risk of bias OR not assessed = 0 P; unclear risk of bias = 0.25P)
[2] ≥2/3 of overall score = 2 P; ≥1/3 of overall score = 1 P; otherwise = 0 P
[3] 0-3.49: very low meta-evidence; 3.5-5.49: low meta-evidence; 5.5-6.99: moderate meta-evidence; ≥7: high meta-evidence

NutriGrade scoring system for SRs without MA of cohort studies

1) Risk of bias/ study quality/ study limitations **(2 P)**
   a. No information available (0 P)
   b. Risk of bias (2 P)
      i. Ascertainment of exposure[1]
      ii. Adjusted basic & outcome relevant model[1]
      iii. Assessment of outcome[1]
      iv. Adequacy of follow-up duration[1]
   c. Study quality (2 P)[2]

2) Precision **(1 P)**
   a. <500 events or <2000 participants (0 P)
   b. ≥500 events or ≥2000 participants (1 P)

3) Heterogeneity **(1 P)**
   a. >1/3 of included studies have an inconsistent result (i.e. point estimates and/or 95% CI did not overlap between studies) (0 P)
   b. ≥2/3 of included studies have a consistent result (i.e. point estimates and/or 95% CI did overlap between studies) (1 P)

4) Directness **(1 P)**
   a. Differences in population; differences in intervention; surrogate markers; network meta-analysis (0 P)
   b. No important differences in population or intervention; hard clinical outcome (1 P)

5) Funding bias **(1 P)**
   a. Industry funding OR conflict of interest (0 P)
   b. Private institutions, foundations, non-governmental organizations (0.5 P)
   c. Academic institutions, research institutions (1 P)

6) Effect size **(2 P)**
   a. No effect for >1/3 of included studies (0 P)
   b. Moderate effect size for ≥2/3 of included studies (1 P)
   c. Large effect size for ≥2/3 of included studies (2 P)

Overall Score[3]

95% CI: confidence intervals; P: point(s); RR: risk ratio.

[1] ≥2/3 of studies low risk of bias = 0.5 P; >1/3 of studies high risk of bias OR not assessed = 0 P; unclear risk of bias = 0.25 P)

[2] cut-off for different quality scale (≥3/4 of overall score= 2 P; ≥1/2 of overall score= 1 P; <1/2 of overall score= 0 P); i.e. **Newcastle-Ottawa Scale** (mean): ≥7= 2 P; 4-6.9= 1 P; 0-3.9= 0 P;

[3] 0-2.99: very low meta-evidence; 3-4.49: low meta-evidence; 4.5-5.99: moderate meta-evidence; **≥6: high meta-evidence**
